# Cardiac, renal and uterine hemodynamics changes throughout pregnancy in rats with a prolonged high fat diet from an early age

**DOI:** 10.1371/journal.pone.0234861

**Published:** 2020-06-30

**Authors:** Lidia Oltra, Virginia Reverte, Antonio Tapia, Juan M. Moreno, Francisco J. Salazar, María T. Llinás

**Affiliations:** Department of Physiology, School of Medicine, University of Murcia, Biomedical Research Institute in Murcia (IMIB-Arrixaca), Murcia, Spain; University Medical Center Utrecht, NETHERLANDS

## Abstract

**Objective:**

To examine whether the cardiac, renal and uterine physiological hemodynamic changes during gestation are altered in rats with an early and prolonged exposure to a high fat diet (HFD).

**Methods:**

Arterial pressure and cardiac, renal, uterine and radial arteries hemodynamic changes during gestation were examined in adult SD rats exposed to normal (13%) (n = 8) or high (60%) (n = 8) fat diets from weaning. Plethysmography, high-resolution high-frequency ultrasonography and clearance of an inulin analog were used to evaluate the arterial pressure and hemodynamic changes before and at days 7, 14 and 19 of gestation.

**Results:**

Arterial pressure was higher (P<0.05) in rats with high than in those with normal (NFD) fat diet before pregnancy (123 ±3 and 110 ±3 mmHg, respectively) and only decreased at day 14 of gestation in rats with NFD (98±4 mmHg, P<0.05). A significant increment in stroke volume (42 ±10%) and cardiac output (51 ±12%) was found at day 19 of pregnancy in rats with NFD. The changes in stroke volume and cardiac output were similar in rats with NFD and HFD. When compared to the values obtained before pregnancy, a transitory elevation in renal blood flow was found at day 14 of pregnancy in both groups. However, glomerular filtration rate only increased (P<0.05) in rats with NFD at days 14 (20 ±7%) and 19 (27 ±8%) of gestation. The significant elevations of mean velocity, and velocity time integral throughout gestation in radial (127 ±26% and 111 ±23%, respectively) and uterine (91 ±16% and 111 ±25%, respectively) arteries of rats with NFD were not found in rats with an early and prolonged HFD.

**Summary:**

This study reports novel findings showing that the early and prolonged exposure to a HFD leads to a significant impairment in the renal, uterine and radial arteries hemodynamic changes associated to gestation.

## Introduction

The systemic, renal and uterine hemodynamic changes throughout gestation have been extensively examined [1–5] but the results obtained are very often contradictories because the methods used were not adequate for long-term studies in the same subjects. However, it is accepted that the physiological changes throughout gestation include a decrease in arterial pressure (AP) [1,2], an increase in cardiac output (CO) [3,4], an elevation in glomerular filtration rate (GFR) [1,6] and a progressive increment in the uterine blood flow [5]. These hemodynamic changes are necessary to provide the nutritional needs of the fetus.

Maternal obesity is a health problem with an increasing prevalence worldwide that is associated with an altered cardiovascular adaptation during gestation and adverse pregnancy outcomes [7–9]. However, very little is known on the cardiovascular, renal and uterine hemodynamic adaptations to pregnancy in subjects with a prolonged exposure to a high fat diet (HFD) from an early age. This information is important because the prevalence of childhood overweight is set to rise even more during the next years [10] and obesity induced by overnourishment in adolescents results in major placental restriction during gestation [11]. The main objective of this study was to examine the impact of a prolonged HFD from weaning up to and throughout pregnancy on the AP, cardiac, renal and uterine hemodynamic changes associated to gestation. The hypothesis was that this prolonged HFD would lead to an altered vascular adaptation in organs that contribute to the decrease in total peripheral resistance and adequate fetal development during gestation. This hypothesis is supported by results showing that females with a HFD from weaning are hypertensive, have an elevation in leptin, a decrease in adiponectin levels, and an enhanced infiltration of lymphocytes in the kidney [12]. Cardiac, renal and uterine hemodynamic changes were assessed before and throughout gestation by non-invasive methods, in rats with normal (NFD) or high fat diets. Hemodynamic changes were also examined in radial arteries because they are closer to known sites of vascular pathology in human intrauterine growth restricted (IUGR) placentas [13].

## Materials and methods

### Animals and experimental procedures

Studies were performed, according to the “European Convention for the Protection of Vertebrate Animals used for Experimental and other Scientific Purposes”, in Sprague-Dawley (SD) rats from the Animals Service of the University of Murcia. The University review committee approved the study prior to beginning research (A1320140709) and all efforts were made to minimize animal suffering. Plastic cages (45x34x20 cm) with wood chip bedding and shredded paper were used for housing in a temperature (21 ± 1ºC) and humidity (45–60% relative humidity) controlled room on a 12/12 light-dark cycle, with ad libitum access to water and food. Female rats of 12–14 weeks of age were paired with fertile males overnight and mating was confirmed when sperm was found in the vaginal smear. Pregnant female rats were then individually housed during gestation and labour. Litter were kept with the mother until weaning. At this point, two female pups were selected and randomly assigned to receive either a NFD or a HFD from weaning to the end of gestation. The remaining male and female pups were used for other studies. Four female rats with each fat diet were housed in each cage from weaning to 13–14 weeks of age. The calories in NFD (Tekland 2014, Energy density: 2.9 Kcal/g) are from proteins (20%), fat (soybean oil) (13%) and carbohydrate (67%). The calories in HFD (Tekland TD.06414, Energy density: 5.1 Kcal/g) are from proteins (18,4%), fat (lard + soybean oil) (60.3%) and carbohydrate (21.3%). Food intake was not measured but a previous study [12] showed that food intake was greater (p<0.05) in rats with NFD (15,3 ± 1,1 grams/day) than in rats with HFD (11,8 ± 1,4 grams/day) at 3,5 months of age.

Female rats with NFD (n = 8) or with HFD (n = 8) were placed with a fertile male overnight at 13–14 weeks of age, and day 0 of pregnancy was considered as the morning that sperm was found in the vaginal smear. Pregnant rats were pair housed up to day 17 of gestation and caged individually four days before labour. Changes in systolic AP (SAP), CO, renal blood flow (RBF), GFR, and uterine and radial arteries hemodynamic were examined before and during pregnancy in both groups of rats with NFD or HFD. Ultrasounds and SAP measurements were performed during less than 30 min in anesthetized rats (isoflurane in O_2_ with the use of a face mask: 4% to induce; 2–2,5% to maintain) on a heated platform to maintain rectal temperature at 37ºC. Arterial pressure was measured by the tail-cuff method (CODA, Kent Scientific, CT) under superficial anesthesia to avoid the stress during the inflation-deflation cycles. The average of 10 measurements were taken as SAP value. In previous studies [14], it was found that the SAP values obtained using this method are highly correlated with those obtained in conscious freely moving rats with intra-arterial catheters.

### Ultrasound studies

Cardiac output, RBF and hemodynamic in uterine and radial arteries were evaluated using a high-resolution micro-ultrasound system (Vevo® 3100, VisualSonics, Toronto, Canadá) and two transducers: MX250 (axial resolution: 50 μm; frequency: 25 MHz) and MX400 (axial resolution: 75 μm; frequency: 40 MHz). Data were transferred to an ultrasound image workstation for analysis (Vevo LAB 3.1.1). The highest point of the systolic waveform was taken as the peak systolic velocity (PSV) and the point of the diastolic waveform as the end diastolic velocity (EDV). Both PSV and EDV were measured from at least five consecutive cardiac cycles. Velocity time integral (VTI) was obtained by outlining five consecutive heartbeat cycles and the integral under the resulting curve was calculated. The time-average velocity (TAV) was measured by the ultrasound system considering the heartbeat cycles.

#### Cardiac hemodynamic

B-mode and M-mode echocardiographic evaluations were performed using the 25 MHz transducer. B-mode was activated to visualize the heart, then M-mode was pressed and measurements of stroke volume (SV), HR and CO obtained from at least three consecutive cardiac cycles.

#### Uterine and radial arteries hemodynamics

Hemodynamic in the uterine and radial arteries were only examined at days 7, 14 and 19 of gestation because radial arteries are first clearly visualized by day 7 of pregnancy. B-mode was pressed to locate the bladder and doppler mode was activated to visualize the uterine artery. Ultrasound evaluation of radial arteries was carried out in four embryos in each rat, two from each uterine horn. 40 MHz probe was used at days 7 and 14, and a 25 MHz probe was used at day 19 of gestation.

#### Renal hemodynamic

Blood flow was measured in the left kidney using 40 MHz probe. B-mode was activated to visualize the renal artery and its diameter measured tracing a line between the internal opposite sides of the arterial wall in two frozen images. Five measurements were obtained in each image to get the arterial diameter. The RBF was calculated from: RBF = HR x VTI x πr^2^, where r is the vessel radius.

### Measurements of GFR in conscious rats

Five hours after the ultrasound and AP measurements finished, GFR was obtained by the transcutaneous measurement of the elimination kinetic of an inulin analog (flurescein-isothiocynate-labelled [FITC] sinistrin) [15]. Rats were anesthetized with isoflurane (2.5%) and a miniaturized device (Manheim Pharma & Diagnostic, Germany) was fixed on a hairless region of the back. Then, a FITC-sinistrin solution (40 mg/ml) was injected trough the tail vein (5 mg/100 g bw) and rats were quickly recovered from anesthesia. Once the recording period (120 min) was over, the device was removed and connected to a PC to download the data. The software provided displays the FITC-sinistrin half-life (t1/2) along with an R2 value. The FITC-sinistrin half-life allows calculating GFR by using a conversion factor (31.26/t1/2). This method is sensitive [15], and does not have some inconvenient of the inulin clearance, such as the requirement of implanting one catheter for blood sampling. In addition, clearance methods generally require restraining the movement and are not adequate during pregnancy because of the risk of incomplete urine collection.

### Statistical analysis

Data in text and figures are given as means ± SE. Data were analyzed using GraphPad Prism 6 software. Differences between experimental periods within one group were evaluated using one way ANOVA for repeated measures with Tukey’s post hoc analysis. Differences between groups were assessed with the use of two way ANOVA and Sidak’s test. Student´s t test were used to examine the differences with respect to the values found during the prepregnancy period within one group (paired) and between groups (un-paired). Two-sided P value lower than 0.05 was considered significant.

## Results

### Body weight and SAP

**Fig 1:**
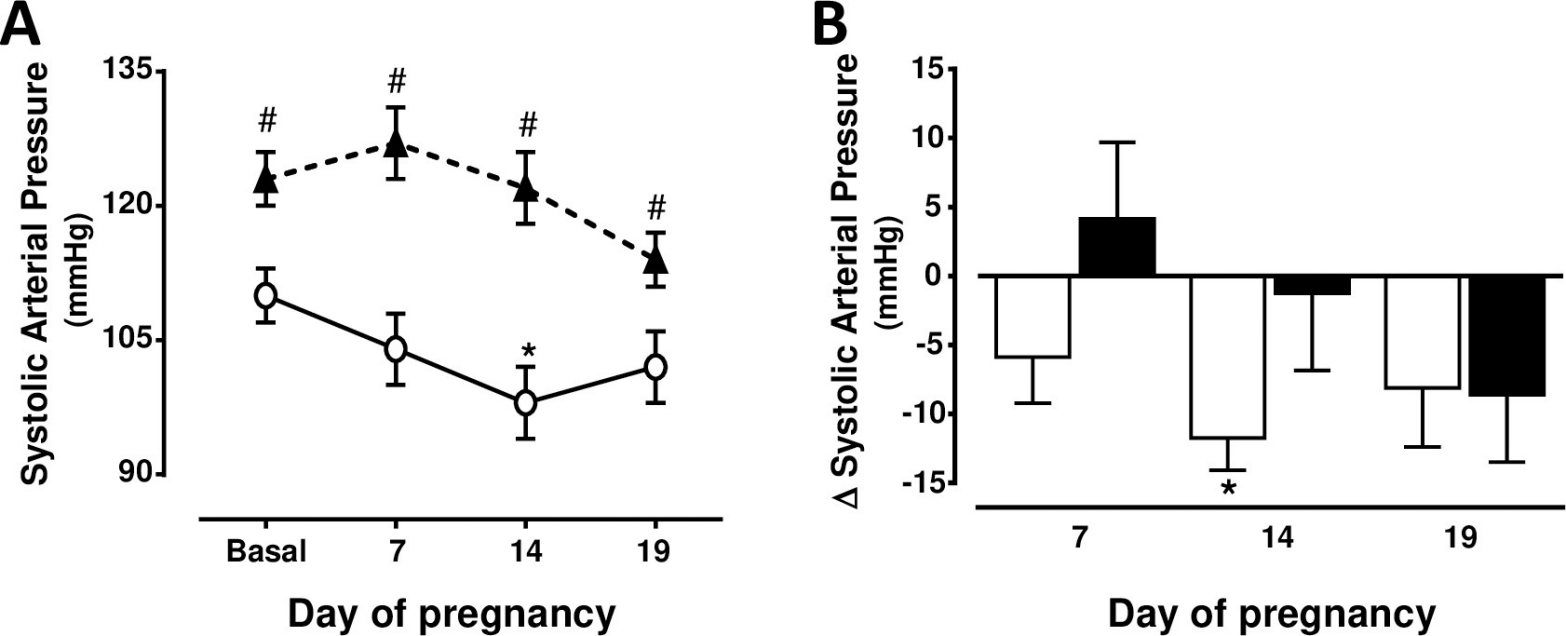
(A) Systolic arterial pressure changes from basal period (prepregnancy) to days 7, 14 and 19 of pregnancy in rats with normal (n = 8) (white circles, continuous line) or high (n = 8) (black triangles, discontinuous line) fat diet from weaning to and throughout gestation. * P<0.05 vs. basal period (ANOVA for repeated measures and Tukey´s test). # P<0.05 vs. normal fat diet (ANOVA and Sidak´s test). (B) Changes with respect to basal period at days 7, 14 and 19 of pregnancy in rats with normal (white bars) or high (black bars) fat diet. * Two-sided P<0.05 within the same group (Student´s paired t test).

Body weight was greater (P<0.05) in rats with HFD than in those with NFD before pregnancy (274 ± 6 g and 237 ± 5 g, respectively) and at day 19 of gestation (355 ± 9 g and 326 ± 12 g, respectively). Increments of body weight were similar in both groups throughout gestation. Fig 1 shows that SAP decreased (P<0.05) in rats with NFD from 110 ± 3 to 98 ± 4 mmHg at day 14 of pregnancy. Before pregnancy, SAP was enhanced (P<0.05) in rats with HFD (124 ± 4 mmHg) and remained elevated (P<0.05) throughout gestation, when compared to the values found in rats with NFD. Considering the changes of SAP during gestation as a whole, they were similar in rats with NFD or HFD (Fig 1).

### Cardiac output, HR and SV

**Fig 2:**
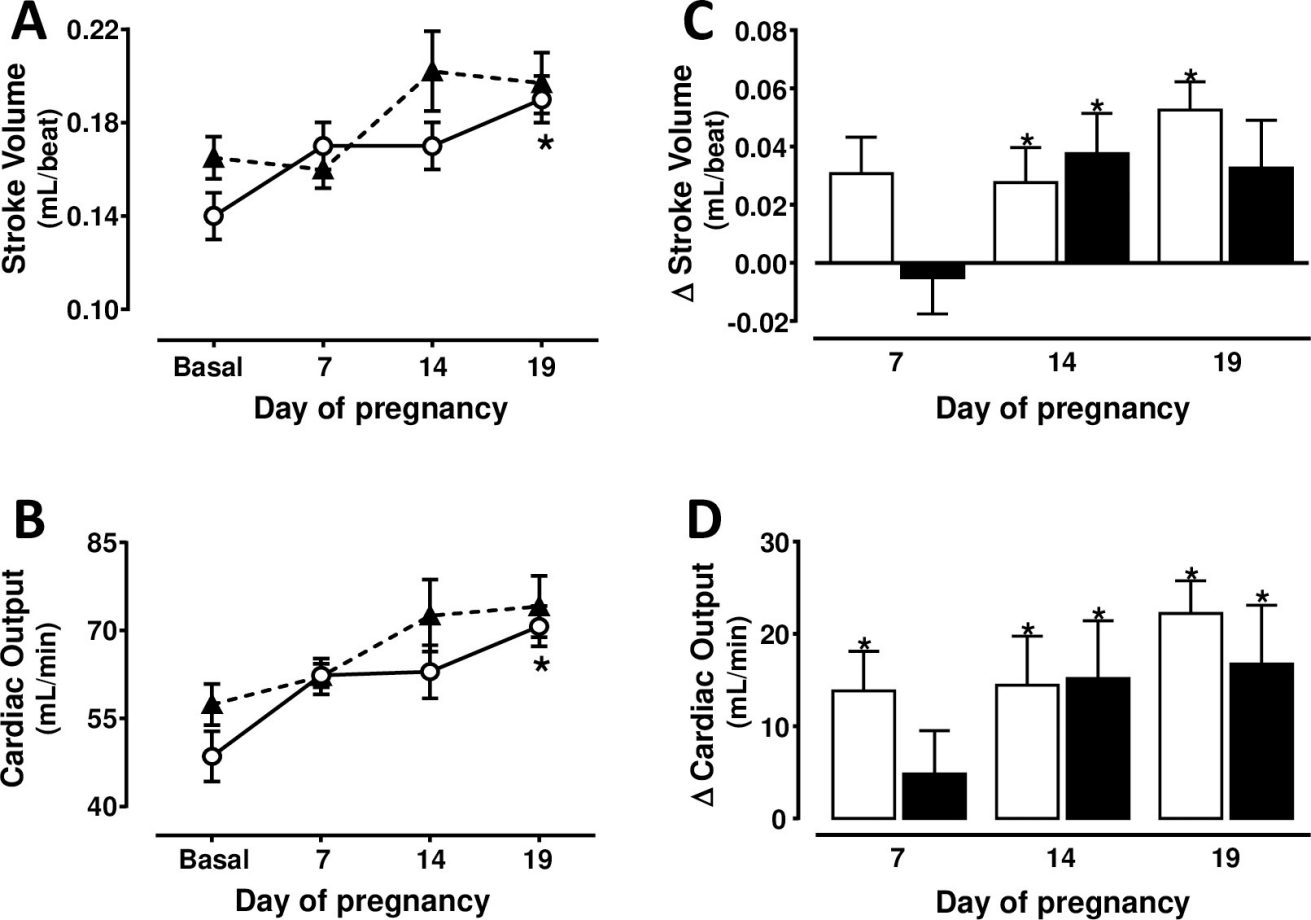
Stroke volume (A) and cardiac output (B) changes from basal period (prepregnancy) to days 7, 14 and 19 of pregnancy in rats with normal (n = 8) (white circles, continuous line) or high (n = 8) (black triangles, discontinuous line) fat diet from weaning to and throughout gestation. * P<0.05 vs. basal period (ANOVA for repeated measures and Tukey´s test). Figures C and D show the changes with respect to basal period in rats with normal (white bars) or high (black bars) fat diet. * Two-sided P<0.05 within the same group (Student´s paired t test).

Cardiac output increased (P<0.05) in rats with NFD from 49 ± 4 ml/min to 71 ± 3 ml/min at day 19 of gestation (Fig 2). This CO change seems to be secondary to a 42 ± 10% increment in SV (Fig 2). An increase in HR (P<0.05) was only found in rats with NFD at day 14 of gestation (377 ± 8 vs 346 ± 9 beats/min before pregnancy). CO was similar before pregnancy in rats with NFD or HFD (Fig 2). The absence of a difference between the CO found in both groups before pregnancy is more evident when their bw (20 ± 2 ml/min/100 g in rats with NFD, and 21 ± 2 ml/min/100 g in rats with HFD) is considered. Taken as a whole throughout gestation, the SV and CO were similar in rats with NFD and with a prolonged exposure to a HFD (Fig 2).

### Uterine and radial arteries hemodynamic

**Fig 3:**
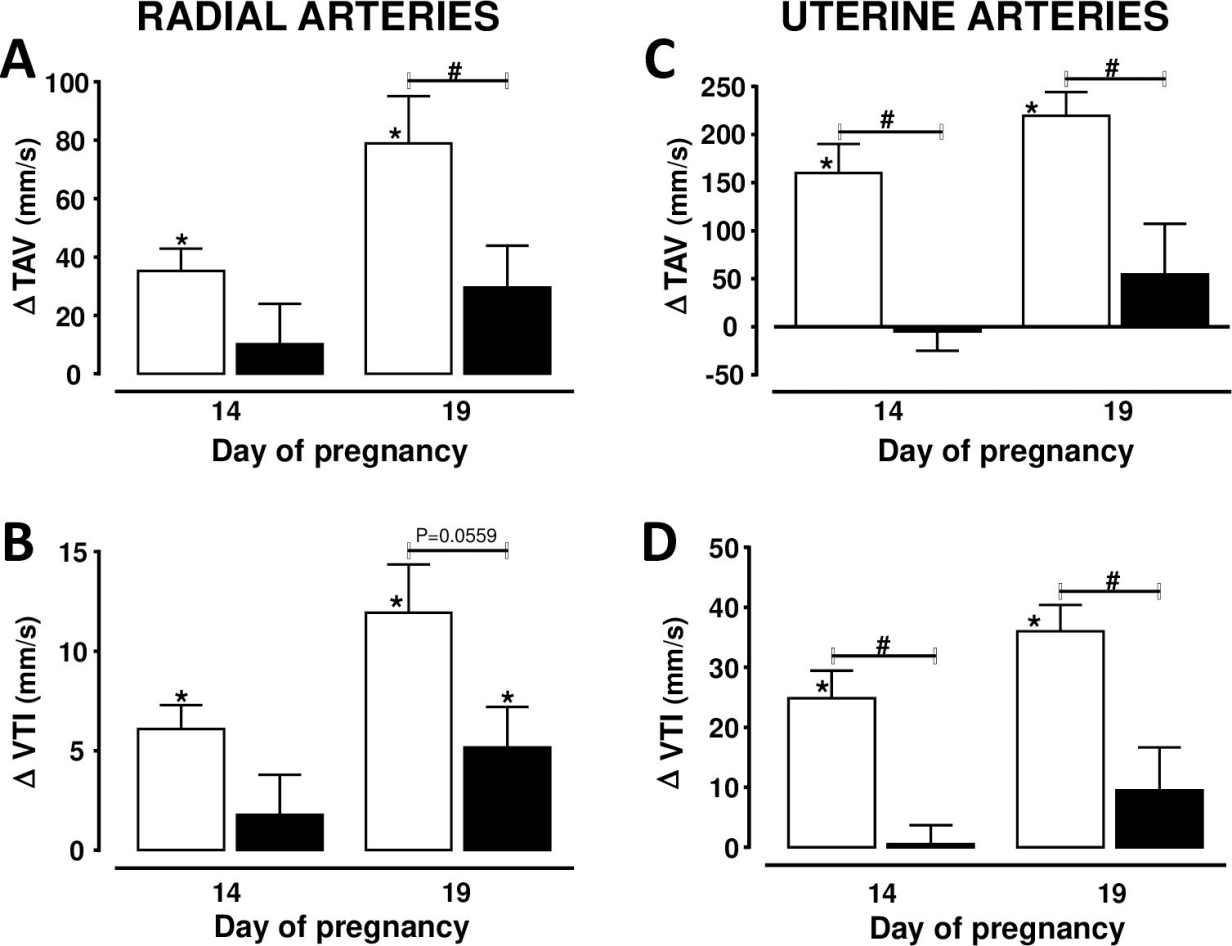
Changes in time average velocity (TAV) and velocity time integral (VTI) in radial (A and B) and uterine (C and D) arteries with respect to day 7 of pregnancy in rats with normal (n = 8) (white bars) or high (n = 7) (black bars) fat diet from weaning to and throughout gestation. * Two-sided P<0.05 within the same group (Student´s paired t test). # Two-sided P<0.05 between both groups (Student´s un-paired t test).

**Fig 4:**
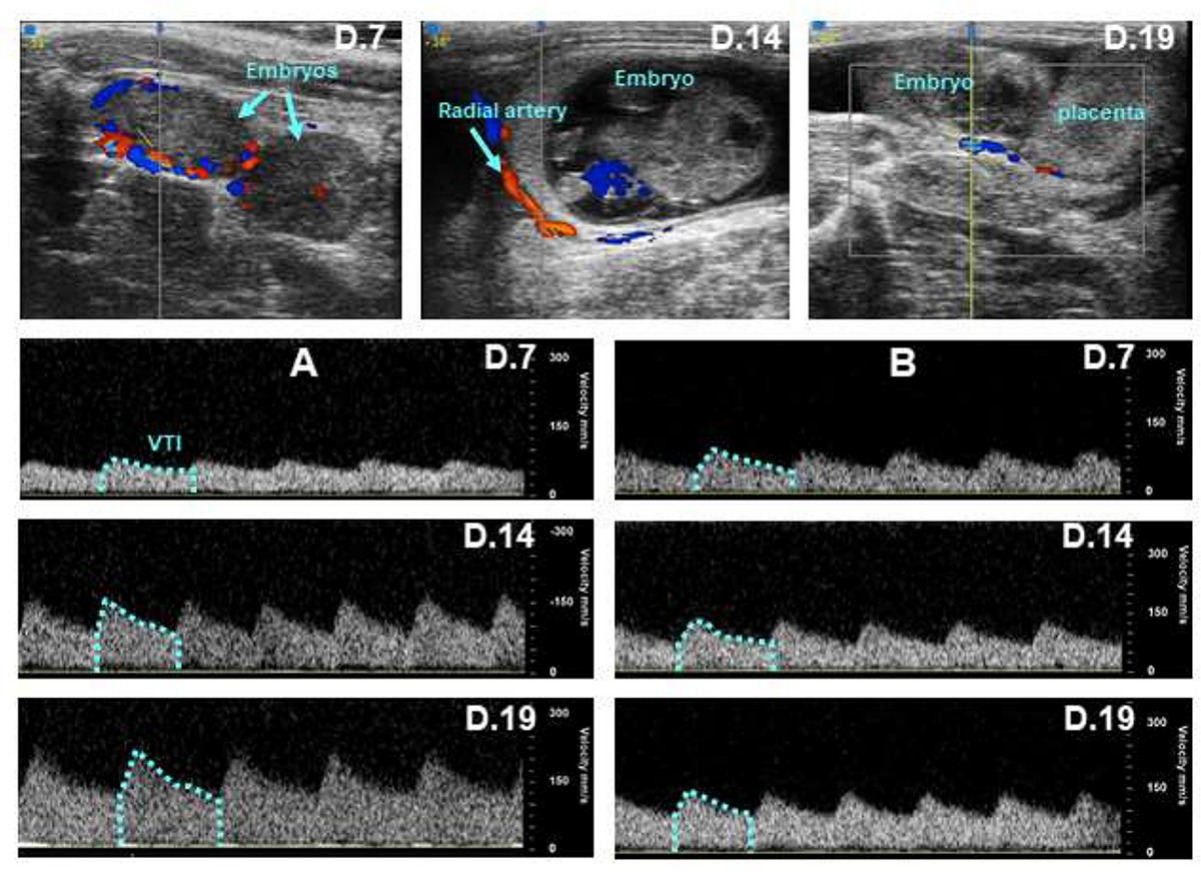
The three images in the upper part show the localization of radial arteries by color Doppler at days 7, 14 and 19 of gestation. The waveforms were obtained in the radial arteries of rats with normal (A) or high (B) fat diet at days 7, 14 and 19 of gestation. The area under the blue lines allows to calculate the velocity time integral (VTI) in each waveform.

Significant hemodynamic changes in the uterine and radial arteries were found in rats with NFD (Fig 3). TAV in the uterine artery increased (P<0.05) from 263 ± 20 mm/s at day 7 to 423 ± 18 mm/s at day 14, and 483 ± 17 mm/s at day 19 of pregnancy. The elevations in PSV and EDV throughout gestation were similar to those of TAV in rats with NFD. Significant changes of VTI were also found in uterine arteries since it increased (P<0.05) at days 14 (80 ± 15%) and 19 (111 ± 25%), with respect to the values found at day 7 of pregnancy. TAV increased (P<0.05) in the radial arteries from 64 ± 3 mm/s at day 7, to 99 ± 7 and 143 ± 16 mm/s at days 14 and 19, respectively (Fig 3). A significant elevation of VTI was also found in the radial arteries of rats with NFD since it increased from 11 ± 1 mm/s at day 7, to 17 ± 1 and 23 ± 3 mm/s at days 14 and 19 of gestation, respectively (Figs 3 and 4). Contrary to what found in rats with NFD, the hemodynamic parameters measured in the main uterine and radial arteries did not change significantly during gestation in rats with a prolonged HFD. Figs 3 and 4 show that TAV and VTI in radial arteries of HFD did not change from day 7 to days 14 and 19 of gestation. EDV in radial arteries was also similar at days 7 (60 ± 6 mm/s), 14 (69 ± 8 mm/s) and 19 (74 ± 11 mm/s) of gestation in HFD rats.

### Renal hemodynamics

**Fig 5:**
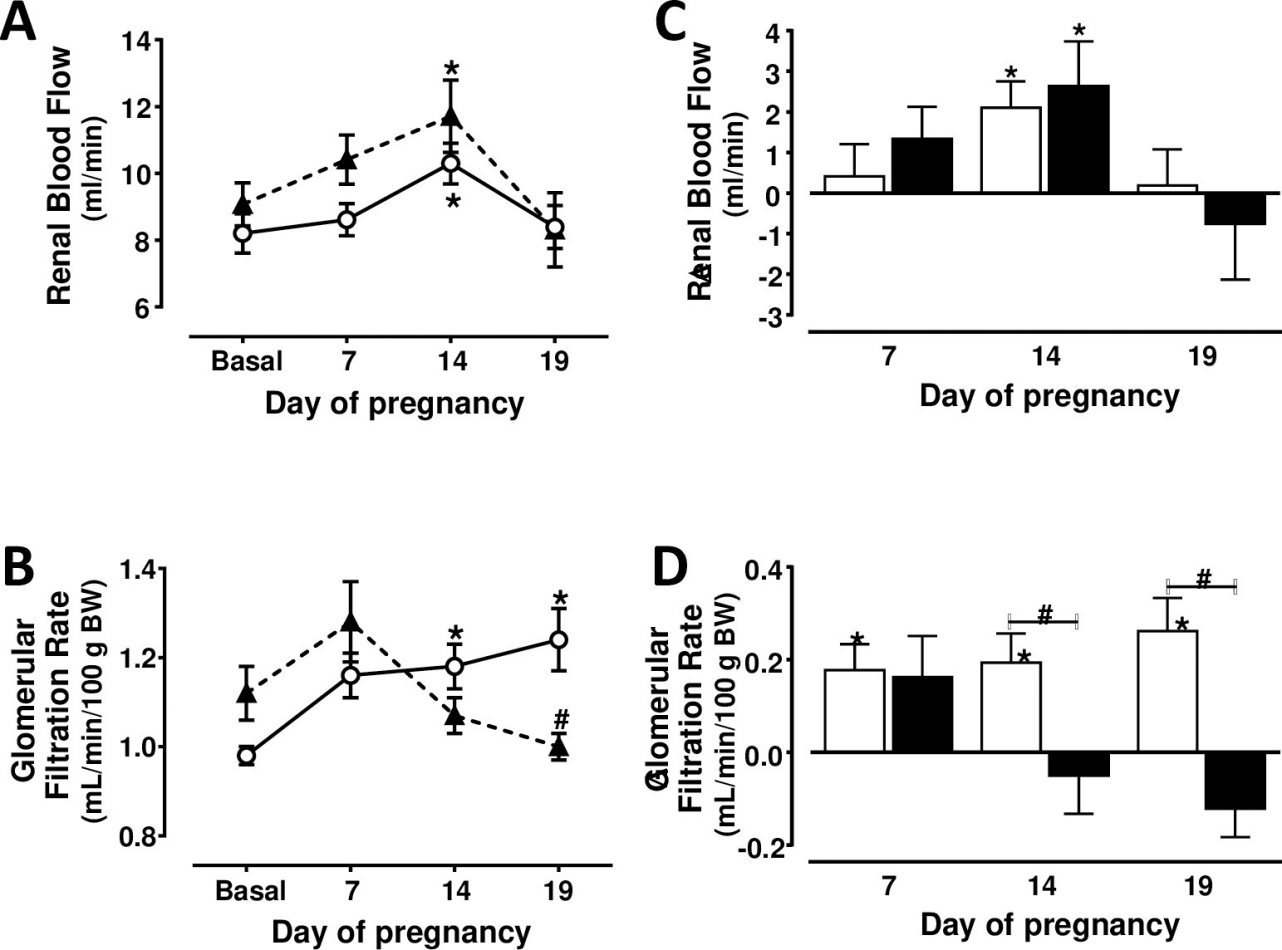
Renal blood flow (A) and glomerular filtration rate (B) changes from basal period (prepregnancy) to days 7, 14 and 19 of pregnancy in rats with normal (n = 8) (white circles, continuous line) or high (n = 8) (black triangles, discontinuous line) fat diet from weaning to and throughout gestation. * P<0.05 vs. basal period (ANOVA for repeated measures and Tukey´s test). # P<0.05 vs. normal fat diet (ANOVA and Sidak´s test). Figures C and D show the changes with respect to basal period in rats with normal (white bars) or high (black bars) fat diet. * Two-sided P<0.05 within the same group (Student´s paired t test). # Two-sided P<0.05 between both groups (Student´s un-paired t test).

No significant differences in renal hemodynamics were found between both groups before pregnancy (Fig 5). A transitory increase in RBF was found at day 14 of gestation in rats with NFD. However, an elevation of GFR was found in these rats at days 14 (1,18 ± 0,05 ml/min/100 gr bw) and 19 (1,24 ± 0,07 ml/min/100 gr bw), when compared to the GFR before pregnancy (0,98 ± 0,02 ml/min/100 gr bw) (Fig 5). A transitory elevation (P<0.05) in RBF was also found in rats with HFD. No significant changes in GFR occurred at days 14 (1,07 ± 0,04 ml/min/100 gr bw) and 19 (1,00 ± 0,03 ml/min/100 gr bw) of gestation in rats with HFD, when compared to the values found before pregnancy (1,12 ± 0,06 ml/min/100 gr bw). Fig 5 also shows that GFR was greater (P<0.05) in rats with NFD than in rats with HFD at day 19 of gestation.

## Discussion

This study reports new findings showing to what extent the changes in SAP, CO, RBF, GFR and hemodynamic in the uterine and radial arteries are altered during gestation as a consequence of a prolonged exposure to a HFD from weaning. The most notable findings are that the prolonged HFD from an early age leads to a significant impairment in the renal, uterine and radial arteries hemodynamic changes associated to pregnancy. These hemodynamic changes occurred despite body weight was only slightly enhanced in rats with HFD.

The cardiac, renal and uterine hemodynamic changes throughout gestation were evaluated in the same subjects by noninvasive methods. It is important because it provides the opportunity to determine whether the renal and uterine hemodynamic changes during pregnancy are associated to those in cardiac function in subjects with a prolonged HFD. The hemodynamics changes were examined by color Doppler technology with high spatial and temporal resolution that allows to detect structural and hemodynamic changes [16,17].

Although there are contradictory results with respect to the AP changes during pregnancy [1,2], the modest decrease found in rats with NFD is similar to that reported in rodents [18] and women [19]. A decrease in systemic vascular resistance, as a consequence of an increment in NO and relaxin [1,2,20], may be involved not only in the modest reduction of AP but also in the elevation of SV of and CO. The observed changes in SV and CO are also similar to those found in women [3] and mice [4].

This study shows new data evaluating the hemodynamic evolution in radial arteries during pregnancy and using high resolution ultrasound. The continuous increments in VTI and TAV in the uterine and radial arteries are consistent with a progressive rise in blood flow since there is an elevation in the uterine artery diameter during gestation [5]. This change in blood flow suggests that there is a continuous increase in the proportion of blood ejected from the heart to the placenta, which is necessary for the correct placental and every organ fetal growth. This vasodilation has been attributed to several mechanisms such as relaxin, vascular endothelial growth factor, and NO [1,21].

Numerous studies have examined the renal hemodynamic changes during pregnancy but there is a considerable heterogeneity in their findings that may be explained by the use of invasive methods at different gestational periods [1,22,23]. The changes of GFR and RBF in our rats with NFD (Fig 5) are similar to that reported in women [6] during gestation. A continuous increase of GFR was found but RBF only increased at day 14. A decrease in plasma colloid osmotic pressure, an increase in blood volume and increments in relaxin and NO may be involved in the physiological renal hemodynamic changes during pregnancy [6,24–26].

The importance of evaluating the impact of an early and prolonged HFD on the cardiac, renal and uterine hemodynamics changes during pregnancy is obvious because the offspring of dams with an early HFD may develop cardiovascular and metabolic dysfunctions at early ages [27–29]. The SAP elevation before pregnancy in rats with HFD (Fig 1) are associated to increases in fat abdominal volume and leptin, and a decrease in adiponectin [12]. Epidemiological and experimental studies showed that AP is elevated at the middle and at the end of gestation in obese subjects [30] but they did not examine whether AP was already elevated before pregnancy. This study reports novel findings showing that AP does not change significantly throughout gestation in rats with an early and prolonged exposure to a HFD. The similar changes in SV and CO throughout gestation in rats with NFD and those with HFD (Fig 2) suggest that the cardiovascular adaptation to pregnancy is unaltered in overweight subjects.

The absence of significant changes in the uterine and radial arteries hemodynamics suggests that a HFD from an early age leads to an inability of utero-placental blood flow to increase with advancing gestation. Previous studies have examined the hemodynamic changes in the uterine artery during gestation in obese subjects [31,32]. However, there are no studies evaluating to what extent the hemodynamic changes thought the radial arteries are also altered in overweighted subjects with an early and prolonged HFD. It is important since the hemodynamic changes in the radial arteries may affect to a greater extent the blood hemodynamic through the spiral arteries than the hemodynamic changes in the uterine artery. TAV and VTI in the radial arteries were similar in both groups of rats at day 7 of gestation, despite AP was enhanced in rats exposed to a HFD. The absence or delayed normal late-gestational increase of EDV to the placenta has been reported in IUGR mouse models and in human fetuses with IUGR [33,34]. The altered hemodynamic changes are important since an early insult on uteroplacental development precedes the late-gestation reduction in placental mass [35, 36]. Our results are also in accordance with those showing an altered vascular development in the placenta of rats with HFD [37]. The fact that the uterine and radial arteries hemodynamic are affected, suggest that the blood supply to fetal organs is significantly deteriorated during pregnancy in rats fed a HFD early in life. It is important that the effect of the prolonged exposure to a HFD on uterine and radial arteries hemodynamic occurred despite body weight was only slightly elevated before and during pregnancy. These results are in agreement with those reported in nonhuman primates [32], showing that the decrease in uterine blood flow is independent of the obese maternal phenotype.

The consequences of obesity during pregnancy on the offspring renal function have been examined [28,38] but it was unknown to what extent the physiological renal hemodynamic changes are altered during gestation in overweighted subjects with an early and prolonged HFD. This study reports novel findings showing that RBF increases transitorily in rats with a HFD, but in the absence of a decrease in AP. The absence of a significant elevation in GFR during most of gestation in rats with a prolonged HFD (Fig 5) is important since glomerular hyperfiltration allows eliminating the waste products of metabolism and it is consequently necessary to have a healthy pregnancy. The renal hemodynamic changes in rats fed a prolonged HFD may be related to the increments in interleukin-6, infiltration of T cells in the renal tissue, albuminuria and leptin levels, and to the decrease in adiponectin in these rats before pregnancy [12,39]. The reduced uteroplacental flow may also be related to the absence of a significant increment in GFR during the second and third week of pregnancy. The results of this study also suggest that the elevated AP at the end of pregnancy in rats with HFD is not only secondary to the release of vasoactive factors since the difference in AP between both groups rats is similar before and at the end of gestation (Fig 1).

Changes in leptin, adiponectin and inflammatory mediators have been proposed to be involved in the altered uteroplacental blood flow during pregnancy in obese subjects [21,31,40]. Taking together with those reported previously [12], our results suggest that an early and prolonged HFD may alter the intrauterine environment as a consequence of changes in adipokines and inflammatory mediators. An imbalance in the circulating levels of pro- (VEGF and PlGF) and anti-angiogenic (sFlt-1) factors could also be involved in the AP increment and in the altered renal and uterine hemodynamic changes during pregnancy in rats with an early and prolonged exposure to a HFD. The imbalance in these angiogenic factors have been proposed by several authors in preeclampsia but there are fewer studies investigating whether they are modified in overweighted and obese females [41] Further studies evaluating the mechanisms involved in the renal and uterine hemodynamic dysfunctions found in overweighted subjects with HFD are needed.

In summary, this study reports new data showing that overweight females with an early and prolonged HFD have a significant impairment in the renal, uterine and radial arteries hemodynamic changes associated to gestation. New data are also reported showing that the changes in the hemodynamic through the renal, uterine and radial arteries are not secondary to a decrease in CO. Further studies are also needed to examine to what extent these hemodynamic changes contribute to the development of cardiovascular and metabolic dysfunctions in the progeny. To determine the mechanisms involved in this altered renal and uterine hemodynamic adaptation throughout gestation is of crucial importance because an altered blood supply to the placenta leads to depressed maturation and proliferation of working cardiomyocytes in the fetal heart [42]. The reduction in uterine blood flow also enhances the risk to adult cardiovascular and renal diseases [43], even in offspring with birthweights within the normal range [10].

## Supporting information

S1 Data (XLS)
